# *Molecular Systems Biology* at 20: reflecting on the past, envisioning the future

**DOI:** 10.1038/s44320-025-00170-w

**Published:** 2025-11-20

**Authors:** Poonam Bheda, Jingyi Hou, Ruedi Aebersold, Uri Alon, Joel S Bader, Lee Bardwell, Edison T Liu, James C W Locke, Matthias Mann, Andrew J Millar, Felix Naef, Yitzhak Pilpel, Ron Shamir, Dennis Vitkup

**Affiliations:** 1 EMBO, Heidelberg, Germany; 2 Institute of Molecular Systems Biology, ETH Zürich, Zurich, Switzerland; 3 Sagol Institute for Longevity Research, Weizmann Institute of Science, Rehovot, Israel; 4 Institute for Computational Medicine and Department of Biomedical Engineering, Johns Hopkins University, Baltimore, MD USA; 5 Charlie Dunlop School of Biological Sciences, University of California, Irvine, CA USA; 6 The Jackson Laboratory, Farmington, CT USA; 7 Sainsbury Laboratory, University of Cambridge, Cambridge, UK; 8 Max Planck Institute Biochemistry, Martinsried, Germany; 9 Novo Nordisk Foundation Center for Protein Research, Department of Cellular and Molecular Medicine, Faculty of Health and Medical Sciences, University of Copenhagen, Copenhagen, Denmark; 10 Institute for Quantitative Biology, Biochemistry and Biotechnology (IQB3), University of Edinburgh, Edinburgh, UK; 11 Institute of Bioengineering, School of Life Sciences, Ecole Polytechnique Fédérale de Lausanne (EPFL), Lausanne, Switzerland; 12 Weizmann Institute of Science, Rehovot, Israel; 13 The Blavatnik School of Computer Science and AI, Tel Aviv University, Tel Aviv, Israel; 14 Department of Systems Biology and Department of Biomedical Informatics, Columbia University, New York, NY USA

**Keywords:** Biotechnology & Synthetic Biology, Computational Biology, Methods & Resources

## Abstract

In this Editorial commemorating the 20th anniversary of Molecular Systems Biology, authors from the inaugural issue in 2005 reflect on how systems biology has evolved since then and share their perspectives on where it is headed in the future.

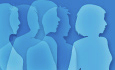

## Teaser

As *Molecular Systems Biology* marks its 20th anniversary, we take this moment to reflect on two decades of discovery and innovation. Since its launch, the journal has stood at the forefront of integrating quantitative biology, computational modeling, and systems science—helping to shape how we understand complex biological systems.

Looking ahead, *Molecular Systems Biology* remains committed to championing systems-level thinking, methodological rigor, interdisciplinary research, and open science. The next decades promise unprecedented opportunities: from harnessing AI-driven insights to exploring systems-level principles of health and disease and ultimately toward realizing predictive biology and medicine across molecular and physiological scales.

In celebration of our 20th anniversary, we reached out to authors from our inaugural 2005 issue to reflect on that first issue and how their own fields and systems biology as a whole have evolved, and to share their perspectives on its future. We are excited to share their thoughts and look forward to continuing to support scientific discoveries from this community, while promoting the integration of systems biology perspectives across the full spectrum of life sciences research.

## Ruedi Aebersold: the first 20 years of *MSB* were grand; The next 20 years will be grander

**Figure:**
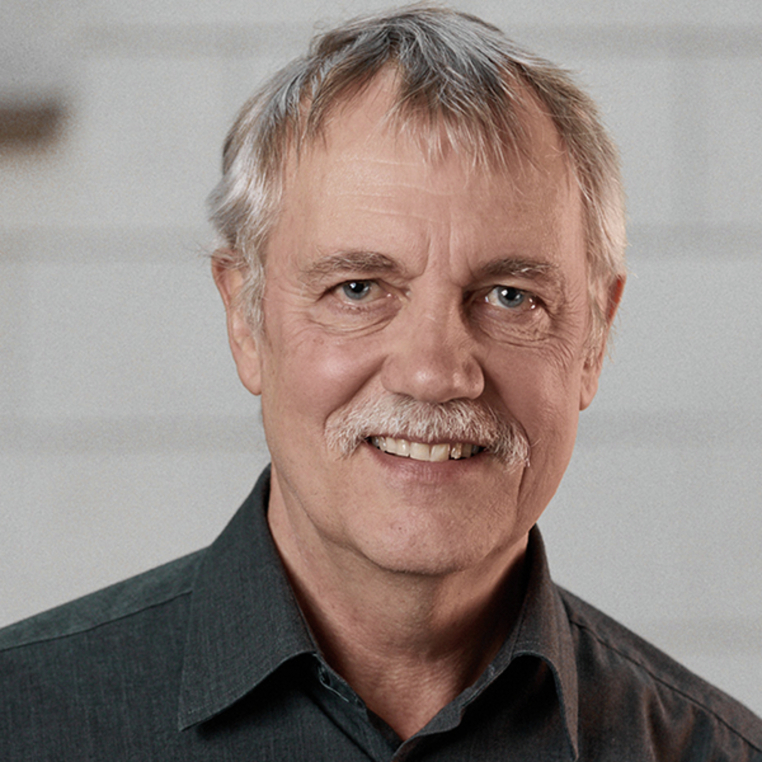

Congratulations, *MSB* on the first 20 years of existence, on becoming a premier venue for publishing groundbreaking systems biology research, and on embracing and innovating new avenues in scientific review and publishing.

After its inception, I had the pleasure of serving as senior editor of the journal for more than a decade. Following the trajectory of the field and arguments ventured below, I predict that the next decades will be the golden age of systems biology because a systems-level consideration of biological processes appears ever more feasible and indispensable.

Systems biology in its present manifestation emerged because of technical developments started in the 1980s that aimed at comprehensively measuring the molecular makeup of biological samples. Following the initial focus on the genome, the “omics” toolbox quickly grew by the inclusion of transcriptomics, proteomics, metabolomics, and more. The ensuing availability of - frequently openly accessible - datasets highlighted the challenge of translating data into new biological or medical knowledge. *MSB* should be commended for publishing foundational technical and computational advances, datasets, as well as new insights into the structure and function of biological systems, thus chronicling in its volumes the whole breadth of systems biology evolution. The following is a somewhat subjective recount of this period with a speculative outlook. Because every field of science needs a conceptual framework of shared beliefs and assumptions, I segment the twenty years into three phases according to the evolution of the prevailing framework.

### Phase 1: systems biology as high-throughput molecular biology

For most of the 20th century biology has consisted of two broad approaches, genetics and biochemistry. They have in common that they attempt to associate observable events with specific molecules, genes and proteins, respectively. The biochemical processes of transcription and translation and the one gene-one protein-one function hypothesis linked the two approaches. This molecule-centered framework was enormously successful and provided a key hypothesis of the genome project: if one knew all genes and therefore all proteins and their functions, one would get closer to understanding living systems.

In reality, the large volumes of data generated by the increasingly powerful omics technologies confounded biologists and suggested that the relationship between genetic variants, gene and protein expression patterns, and phenotypes was too complex to be reduced to specific molecules and functions. Success stories of developing predictive models of the behavior of confined, but nevertheless multimolecular processes, indicated that mathematical models of the process were capable of discovering new biological insights not apparent from knowing the relevant molecules alone. Unfortunately, the modeling strategies do not scale to more complex systems. In summary, the period of systems biology as high-throughput molecular biology very successfully generated unprecedented data resources but was limited by both, the conceptual focus on molecules and the lack of scalable methods for integrative data analysis and modeling.

### Phase 2: systems biology as network biology

Subsequently, molecular networks emerged as a generic and fitting representation of molecules as well as their ordering and relationships. Inference of interaction networks from data from two or more “omics” technologies or direct measurement of molecular interactions generated a range of network types, including protein-protein interaction (PPI) networks, transcriptional networks, kinase-substrate interaction networks, expression-, protein- or phospho QTL networks. These networks turned out to be highly valuable in mapping out the observable interaction space and therefore massively reduced the enormous potential interaction space to those events allowed by evolutionary constraints. Some of these networks, especially when highly curated, are among the most widely used data resources in molecular biology. Nevertheless, they are of limited use to explain the complexities of living systems, for the following reasons: (i) different types of interaction are represented as discrete networks. In the reality of a living cell, different networks are concurrently present and interlinked. (ii) For the most part, networks have been static representations, therefore missing context-specific interaction information, (iii) networks generally do not differentiate between mutually dependent and mutually exclusive interactions guiding the formation of specific functional modules, and (iv) the nodes in network representations are generally invariant.

This phase highlighted the importance and interdependence of molecular interactions as part of the syntax of life, but was challenged to associate the network state with biological context, to reduce the network representation to discrete biochemical modules catalyzing and controlling molecular processes, and to generally explain the interdependencies of molecular mechanisms.

### Phase 3: systems biology as the study of adaptive complex systems (CAS)

In the 1960s sociologist Walter Buckley coined the term “complex adaptive system” and work on the topic and the definition was expanded to include the following major hallmarks: (i) Complexity: The system is composed of a large number of diverse agents, (ii) Adaptability: Agents change in response to signals and feedback, (iii) Connectivity and self-organization: Agents primarily interact with each other in a localized manner via self-organization, (iv) Distributed control. There is no central master organizer and the system adapts via autonomous, decentralized mechanisms, (v) Emergence: System behaviors arise from local interactions, and (vi) Robustness.

Contrasting the CAS hallmarks with the properties of living biological systems, we witness a striking match. Consider a generic signaling system activated when a membrane receptor binds its ligand. This initial event causes the rapid state change of specific “agents” – typically proteins – through phosphorylation, proteolysis, or allosteric binding of small molecules. These activated agents then induce adaptation of the cell’s state by the subsequent, coordinated adaptation of multiple agents in a manner consistent with CAS hallmarks. The initial signal induces selected protein agents to change their state, to locally self-organize into new or altered macromolecular structures that in turn collectively cause the emergence of altered systems behavior. Emergent systems properties include robustness, feedback control, and the phasing out or attenuation of the systems’ response to the initial stimulus. These notions go beyond the “high-throughput molecular biology” or the “network phase” of systems biology. First, the adaptation of individual molecular agents into different states is a central hallmark of CAS based systems biology. Second, the system’s behavior is the consequence of multiple, locally dispersed self-organized adaptations of local interactions. Third, non-linear effects result from the altered molecular interactions rather than abundance. A CAS-centered framework is expected to have significant implications for experimental and computational systems biology because the focus will shift from molecules and discrete networks to adaptive systems, where the adaptation of the state of multiple molecular agents collectively induces the adaptation of the system’s connectivity and behavior.

As stated initially, I am convinced that the next decades will be the golden age of systems biology. This optimism derives from the ongoing extension of the multi(prote)omics toolbox towards the measurement of alterations in the state of the molecular agents and systems behavior, and the emergence of the CAS framework as a guide to technological advances and experimental design in systems biology. I wish the journal *MSB* and the community that supports it great success on this journey.

## Uri Alon: perspective on systems aging, physics, and AI

Aging research is one of the most rapidly growing frontiers in biology. Evolutionary theory in the 1950s predicted there should be no easy way to extend lifespan in organisms. A major turning point in the 1990s was the discovery that single-gene mutations can extend lifespan in worms or yeast, many of which are similar in other organisms such as flies and mice.

### Physics of aging

I’m writing this from the perspective of a systems biologist with a physics background. A brand-new field has been born in the past 2 years, we call the physics of aging.

A small group of physicists working independently in different countries converged on similar stochastic differential equations to describe the accumulation of damage. Our own model, called the saturation removal model, seems to capture many quantitative facts of aging with few parameters. The equations show how damage accumulation causes the well-known patterns of aging: an exponential rise in the risk of death with age, an exponential rise in the risk of diseases with age, and a linear decline in physiological function with age.

Such equations provide a much-needed theoretical basis that can guide research—for example, which interventions are likely to compress morbidity (the time that an individual is sick at the end of life) while extending the lifespan, to translate animal experiments to people, and to understand which drug combinations might work best.

### The role of big data

This decade has also seen a shift from using model organisms like mice to focusing on humans as the organism of interest. There are large datasets on human health. Some are publicly available, such as the UK Biobank and NHANES. Others, like the Israeli 10 K cohort, are designed specifically for machine learning on aging by measuring millions of parameters. Computerized medical datasets like Israel’s Clalit allow researchers to follow millions of people as they age.

### On the future of AI and aging research

An exciting development is the growth in companies and academic researchers aiming to design new geroprotective drugs, not only repurpose existing ones. This combines novel screening methods and novel AI methods to speed the drug discovery pipeline.

AI is also being used to design new small-molecule drugs to bind targets believed to be important for aging. I believe this field will grow as AI learns the language of proteins and small molecules and enables finding novel compounds that are very specific and have minimal crosstalk with other targets.

My students use AI regularly to help them program. I use it to find biological knowledge (this essay however is hand-crafted). There is no doubt that AI will be a useful tool for researchers.

Some biologists believe that an AI foundation model will also be the “answer” eventually in biology. We will feed it with large data and the entire scientific literature. It will then be able to answer any question, like what drug can rejuvenate this liver.

Some are envisioning an automated cycle where AI discovers which experiments are missing that will be most informative, and then runs roboticised, DNA sequencing-based, high-throughput experiments, feeding back to itself and closing the scientific loop. A self-catalyzing cycle of knowledge discovery, with humans out of the equation.

I foresee or wish for an AI-human collaboration. The emphasis will be on training AI to communicate with researchers and explain how it got to its conclusion. The drive will be the esoteric, curiosity-driven questions of a human researcher based on their own unique taste, education, background, and personality. That’s the way science will advance.

## Joel S Bader

Two years into my assistant professor position, what an honor it was to publish one of my first papers in the inaugural issue of *Molecular Systems Biology*. Our paper was motivated by a problem that remains a core challenge of systems biology: we know the parts, but what is the wiring? We can sequence genomes and extract protein-coding genes, but we cannot yet reliably predict how proteins interact with each other, either stably in complexes or transiently as part of regulated signaling networks, to create the emergent behavior of life.

As in Plato’s parable of the cave, where real-world objects are inferred by the shadows they cast on a cave wall, our study attempted to infer mechanistic interactions from genetic interactions. Genetic interactions are loosely defined as genetic perturbations whose combined effect differs from an expectation based on the individual effects. They may have nothing to do with physical interactions in the real world. Our insight was that pairwise genetic interactions (a combined knockout of genes *A* and *B* is lethal, whereas the individual knockouts are viable) could be made more informative by looking for shared patterns of interaction (genes *A* and *B* share many genetic interaction partners in common). Exploiting the awesome power of yeast genetics, we showed that proteins encoded by genes that shared genetic interaction partners were enriched for co-pathway membership and for mechanistic protein-protein interactions. The success of our study, and similar studies by other groups, continues to motivate similar high-throughput genetic perturbation experimental designs, from RNAi and shRNA to CRISPR screens and now to PerturbSeq in mammalian cells.

A second theme at the time was paired progress in genetics and proteomics. Publication of a draft human genome sequence occurred in the same period as publication of high-quality maps of protein-protein interactions and protein complex co-membership by yeast two-hybrid and affinity purification mass spectrometry. Since then, technologies for nucleic acids have had exponential gains. On the proteomics side, we have learned to exploit our growing knowledge of protein interactions with new therapeutic modalities: monoclonal antibodies that target protein surfaces, and PROTACs and molecular glues that create and stabilize protein interactions for therapeutic effect. Computational approaches for physics-based modeling and AI prediction of protein structure and interactions have leapt in performance and continue to advance with incredible speed.

Fundamental technologies for measuring protein-protein interactions at scale, however, have progressed much more slowly than DNA sequencing and computational proteomics. One possible future could be computational inference of protein interactions, followed by wet-lab validations of computational hypotheses. Drug discovery may be on this path, with high-throughput assays replaced by computational design. Even so, computational methods require data for training, and new technologies for protein-protein interactions remain one of the most important challenges for molecular systems biology.

## Lee Bardwell


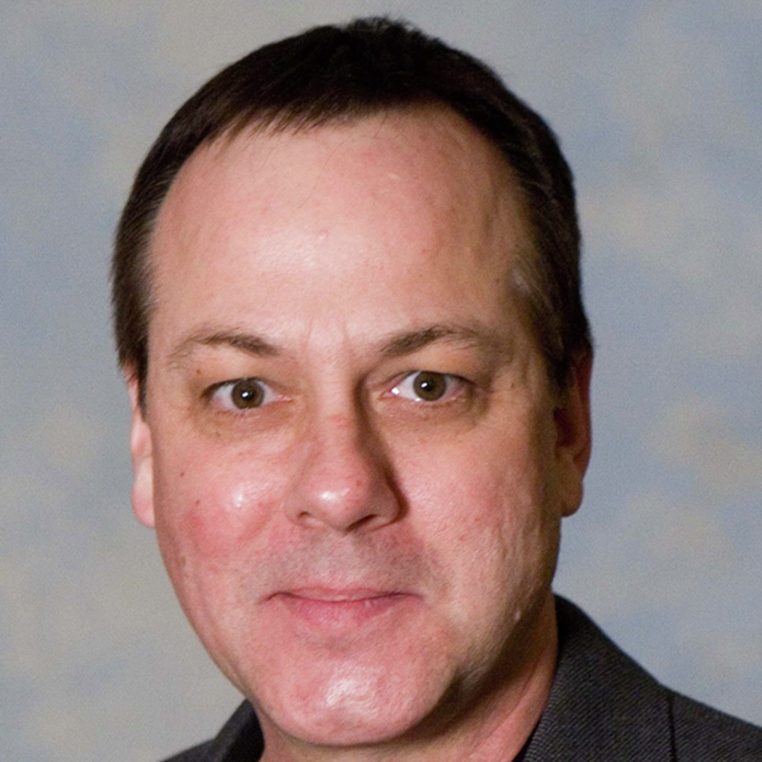


The paper we published in the inaugural issue of *Molecular Systems Biology* was about cellular mechanisms that limit inappropriate crosstalk between different cell signaling pathways. The initial goal of the project was to build a mathematical model of how scaffold proteins promote signaling specificity. We quickly realized, however, that our first task was to devise a formal definition of signaling specificity. We found that a common-sense definition of signaling specificity consisted of two equally important parts, which we termed specificity and fidelity.

We also needed to define the ground state, or basic architecture, of an interconnected signaling network that did not have scaffold proteins or any other “insulating mechanisms”. Ultimately, when we did pare down our scaffolding model and analyzed it using the new definitions, we found that it had a very deep relationship (i.e., very similar equations) to another insulating mechanism, compartmentalization.

The omics explosion of the last 20 years has reinforced the idea that extensive interconnections between different cell regulatory pathways are ubiquitous. My current view is that a lot of the crosstalk that we observe is probably physiologically appropriate signal integration that is supposed to happen in certain tissues at certain times, while some of it is low-level noise that the cell has figured out how to deal with. Beneficial or neutral crosstalk, however, can become pathological in disease states.

When I heard about the new systems biology journal that was a joint venture of *EMBO* and *Nature* publishing, I knew this was where I wanted our study published.

*Molecular Systems Biology* represented the type of interdisciplinary biology we wanted to do: using molecular understanding to build quantitative models that in turn suggested new molecular connections and mechanisms. At the time, there were few dedicated systems biology journals. *Molecular Systems Biology* sounded like a journal that would be read by both experimental biologists and mathematical and computational scientists, and would be a major player in the field going forward. This is exactly what we were looking for. The open-access-only format was also a major attraction for us, as it seemed clear that this would increase the readership and impact of the early papers.

The terms “design principles” and “performance objectives” are possibly overused in systems biology, but for me, the most exciting studies are those that reveal such principles, addressing (for example) not just how a signal gets from the cell surface to the nucleus, but also why the route is so complicated and networked. What other performance objectives shaped the evolution of the network?

Ruedi Aebersold said in the first editorial published in *MSB* that little is known how cells integrate signals from a variety of inputs into a physiological response. Although we have learned a tremendous amount in the last twenty years, I think this is still one of the most important questions in the field.

I believe the editorials and early papers published in *MSB* helped shape the field of systems biology going forward. My colleagues and I are very proud to have played a small part in this.

## Edison T Liu


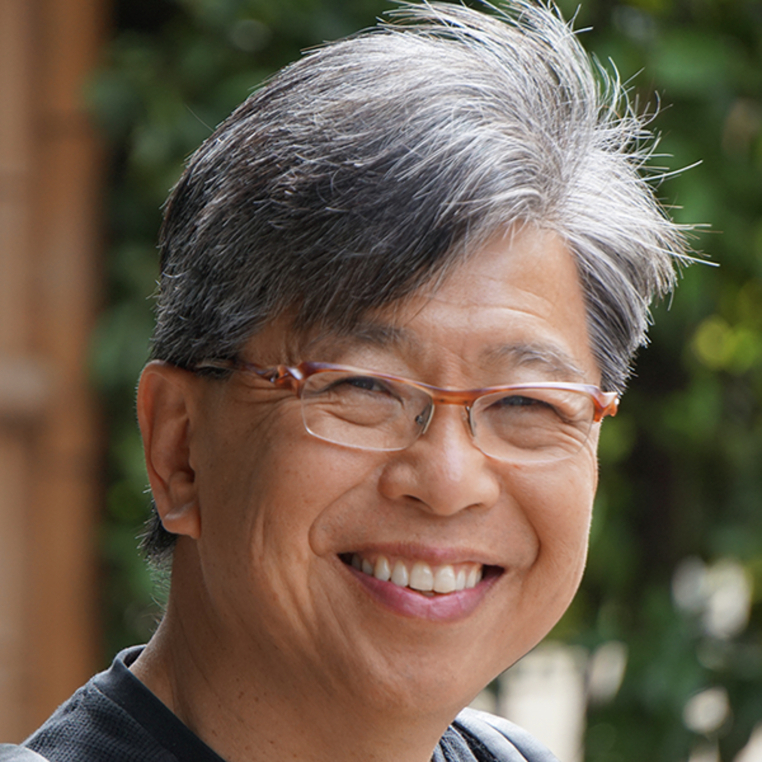


As one of the founding editors for *Molecular Systems Biology*, I was asked in 2005 to give my personal view on the meaning of systems biology to help define this new field for the inaugural issue of the journal. At that time, I stated that systems biology goes beyond explaining biological phenomena on a gene-by-gene basis to interactions of all of the components in a cell or an organism. Operationally, we had to find ways to correctly digitize complex biological data so that we could integrate disparate information into computational working models. The ultimate goal was to achieve predictive biology, i.e., given the list of components involved, to be able to predict de novo the biological processes.

Fast forward 20 years, and computational systems approaches have become the operational norm in nearly all aspects of biological investigations. Importantly, many of these concepts and strategies have been applied to important medical challenges, resulting in a tangible impact on human health. While we have made tremendous progress in building powerful tools for systems analysis of biological problems, we are just at the beginning of the journey to achieve the goal of predictive biology (a.k.a. predictive medicine, precision medicine). Sequence-to-function initiatives, the Chan Zuckerberg Initiative’s Virtual Cells program, medical digital twins (in silico patient) all have been recently launched to create predictive models in biology and medicine. As these models are dynamic, close integration of model construction and experimentation is essential. Once predictive accuracy is achieved, synthetic biology, where new biological processes and even biological entities can be created, may be realized.

## James CW Locke and Andrew J Millar


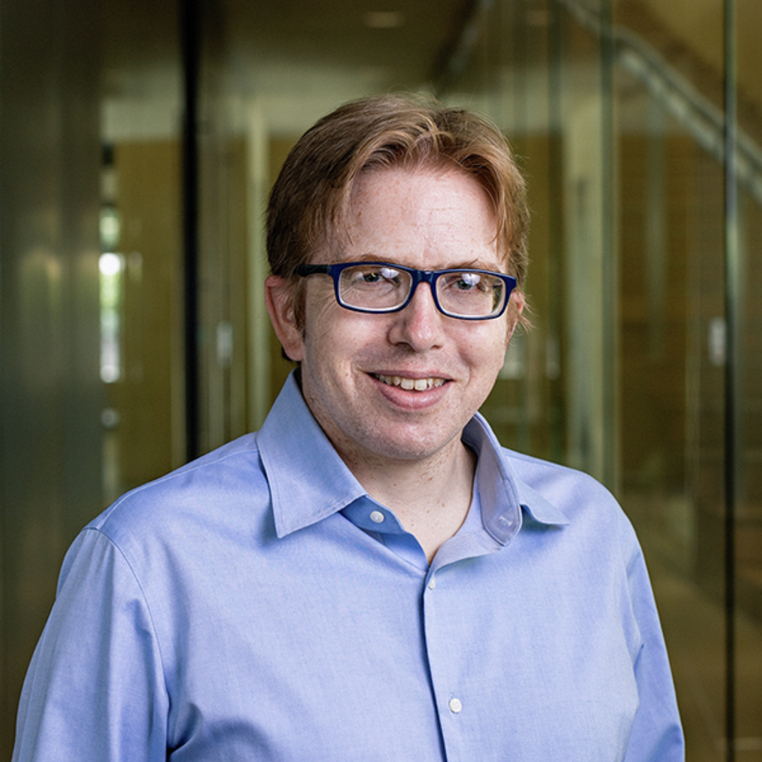

**Figure:**
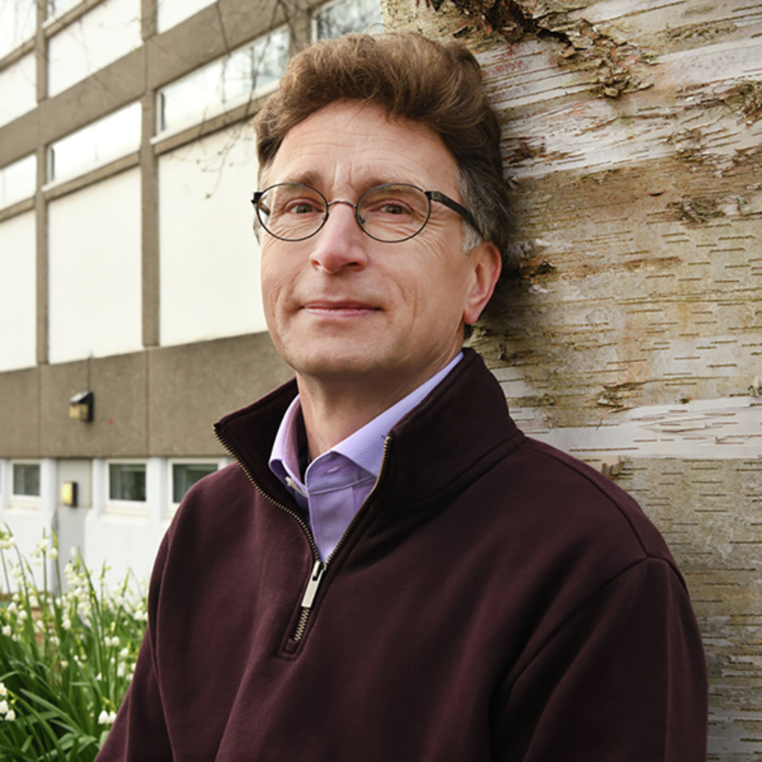
Anne Purkiss (cropped)

Our paper on the circadian clockwork in plants was an early demonstration of the Systems Biology approach in our field, using a model to integrate the insights of many, more focused molecular biology publications, then iteratively proposing and testing new biological functions. The multiple functions that we ascribed to the few components in our model were subsequently revised, as further components that operated in parallel were identified and integrated into a series of more complicated models. Our groups recently used later models to test the spatial coordination of cellular clocks across the plant and incorporated absolute RNA and protein copy numbers per cell with an unexpected link to genome sequence.

The key asset of publishing in *MSB* remains the combination of molecular biology reviewers and editorial expertise, with the mathematical competence that had been neglected by molecular biologists before *MSB* arrived. The Systems Biology approach has still not been adopted widely enough for our research areas to do without this combination.

The discovery frontier is now to understand how biological clocks operate under field conditions and in multiple species, integrating further data types and scientific communities. If we claim to understand the biological clock, then our task is, for this example system, to explain and predict both how genomes in cells build (this aspect of) organisms, and how organisms in populations select (the relevant genes in) their genomes. The growing body of data on single-cell heterogeneity is posing new challenges for understanding the whole-organism phenotype, but the potential for Systems Biology to link up to larger-scale models remains.

## Matthias Mann


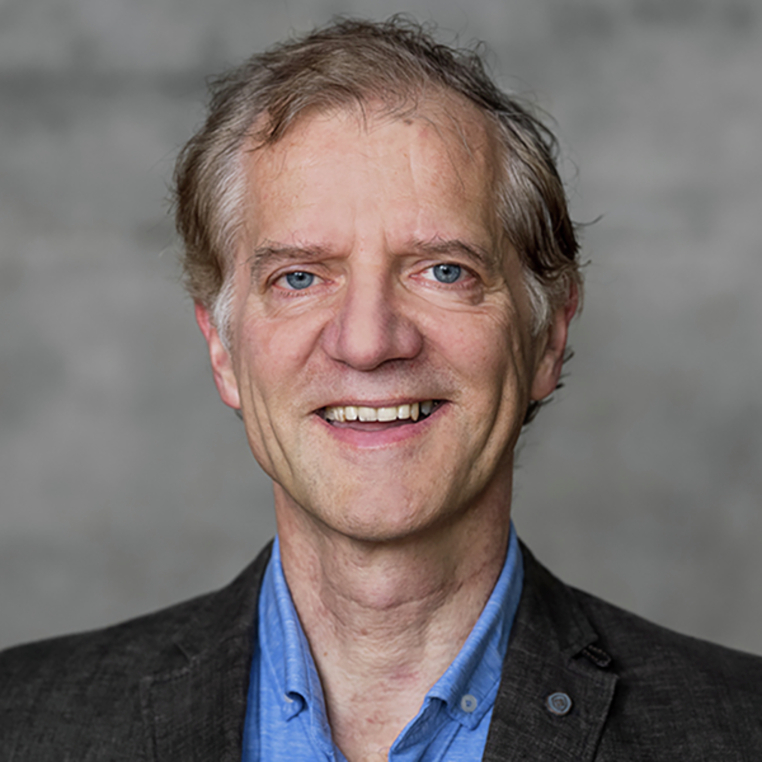


In the inaugural issue of *Molecular Systems Biology*, we reported the first systematic map of a phosphotyrosine interactome using a quantitative mass spectrometry-based pulldown of close to 100 phosphotyrosine peptides covering all ErbB receptor family members. This work demonstrated that proteomics could move beyond isolated signaling events to unravel dynamic networks of interactions, laying a foundation for systems-level analysis of cellular communication.

Two decades later, the principle remains the same, but the scale has expanded by orders of magnitude. What began as a family-level screen has grown into proteome-wide interaction maps—up to entire cellular networks such as the yeast interactome—and is now being extended to more than 15,000 viral proteins in our ongoing effort to chart host–pathogen interfaces. The value of such maps was illustrated during COVID-19, when viral interactomes guided the search for therapeutic vulnerabilities.

AI is already dramatically enhancing the value of MS-derived interactomes. AlphaFold-enabled modeling helps us assign new complex members in yeast and in viral–host assemblies, while new chemical biology-based approaches like PELSA delineate the ligand-sensitive surfaces that mediate binding. These structural interactomes are beginning to yield predictive, intervention-ready models of cellular circuitry.

## Felix Naef


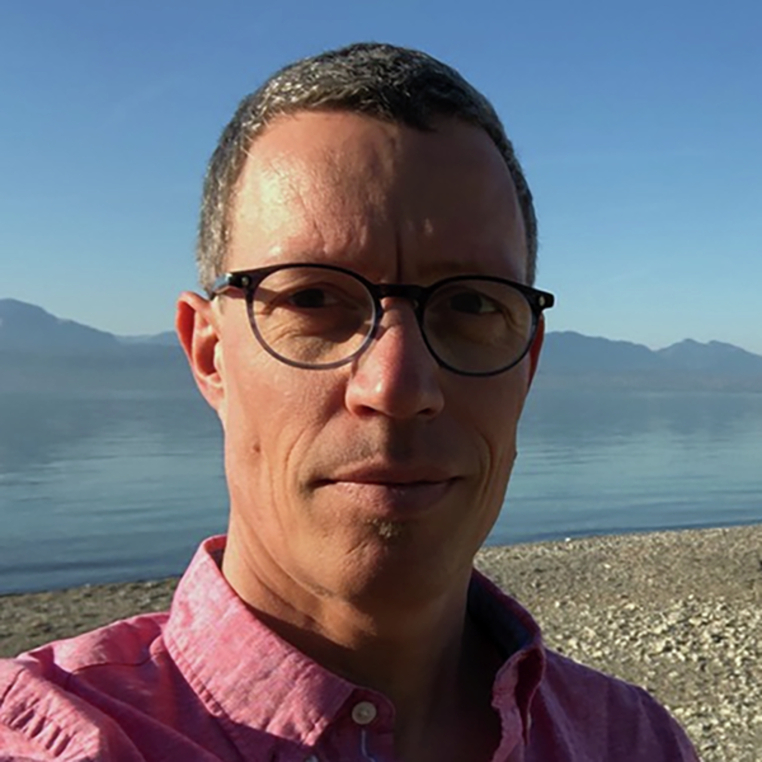


In the inaugural issue of *Molecular Systems Biology*, I published a review on the cyanobacterial circadian clock, focusing on the then-recent discovery that circadian rhythms in Synechococcus can be sustained by purely post-translational oscillations of the KaiC protein. This work was pivotal in highlighting that the clock could be reconstituted in vitro from only three proteins and ATP, establishing cyanobacteria as the best understood circadian model system and providing a reductionist entry point into the molecular mechanics of biological timekeeping.

Over the past 20 years, the field has continued to advance achieving detailed structural and mechanistic understanding of Kai proteins, their dynamic assemblies, and their regulation. New live reporters, including molecular probes and fluorescence anisotropy, now allow circadian protein interactions and states to be visualized in real time. The next frontier lies in bridging this molecular precision with cellular physiology and ecology, to understand how clocks interface with metabolism, stress responses, and organismal fitness in fluctuating environments.

In my view, systems biology thinking is indispensable. Much like systems neuroscience or many-body physics, biological systems are so complex that focusing on single parts, while powerful for dissecting Mendelian traits, cannot explain emergent properties such as robustness, homeostasis, or complex traits. In many ways, physiology has always been a field of systems thinking, even if it was not named that way, and modern systems biology builds on this tradition by combining quantitative models with molecular and genomic data. While systems biology has now diffused across biology, I see value in maintaining systems biology as a perspective and discipline, with the current frontier being to achieve predictive, mechanistic understanding of collective dynamics across molecular and physiological scales.

## Yitzhak Pilpel


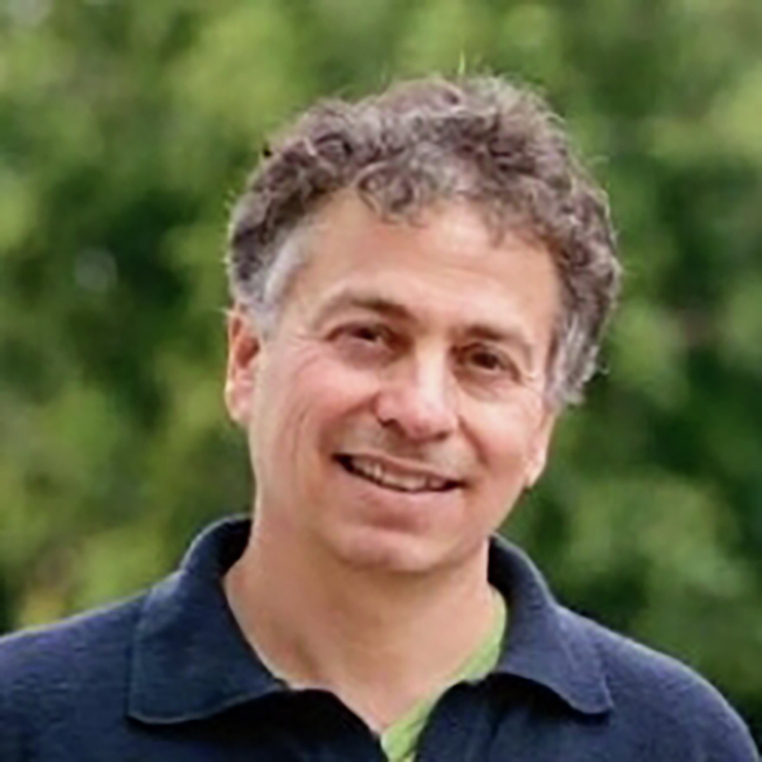


Our paper in the inaugural issue of *Molecular Systems Biology* was based on a pioneering study that employed the method of in-lab evolution, that was and still is, very common in microbes, to human cell lines. Over >600 days, a human cell line was evolved to have cancerous characteristics.

Using microarrays (which was later replaced by RNA-seq) it was possible to ask how the cell transcriptome changed in this process. Our focus was on the regulation of the human cell cycle genes, major drivers of cancerous transformations. It was well known that to commit to cell division, human cells must get multiple green lights (and avoid many red ones). One major channel of information that affects cell division is the cell's internal, and the other is from the cell’s environment. A cell would typically commit to cycling only if the two channels favor this decision. But how do cells “compute” or make decisions based on multiple inputs?

It was already appreciated based on classical studies of gene expression (such as on the lactose operon or phage lambda response in bacteria) that Boolean logic is at work in many biological circuits. Promoters were known to regulate gene expression, computing logical gates such as AND, OR XOR. Our paper was crucial for systems biology because it showed that a complex biological decision-making process, like cell division, is not always based on simple Boolean switches. Instead, it relies on a sophisticated “Analog Gate”. In particular, we found that two continuous signals (one reporting on internal cell state, the other external to the cell) are summed up to produce a proportional, graded output - expression of cell cycle gene and cell doubling time. This put forward the idea that natural genetic networks may function as continuous, analog processing units. This line of investigation also appears in natural and synthetic biological circuits that can perform a plethora of sophisticated arithmetic, not only Boolean logical, computations.

## Ron Shamir


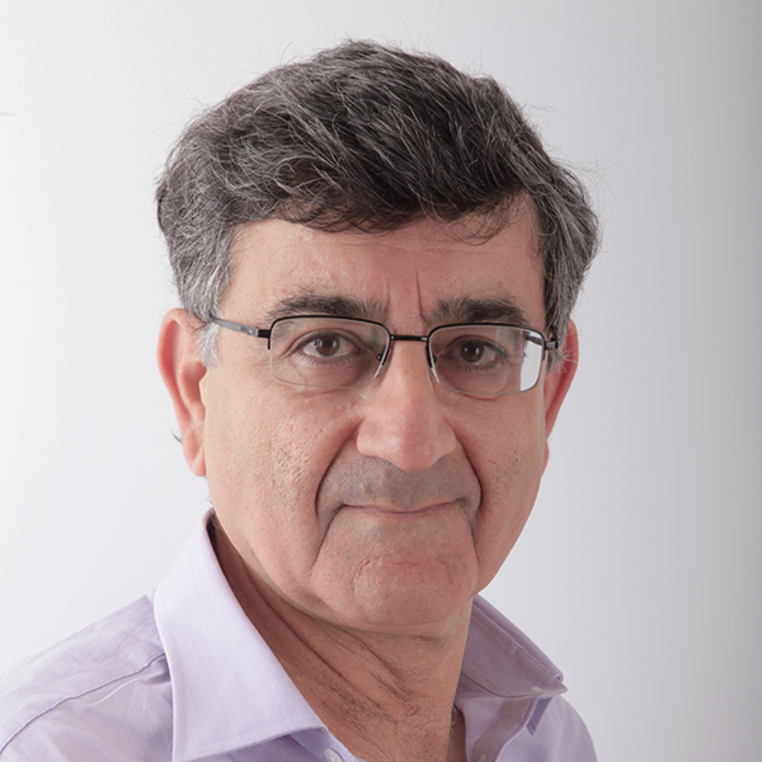


In the inaugural issue of *Molecular Systems Biology*, Amos Tanay, Israel Steinfeld, Martin Kupiec, and I published a study describing a methodology for integrative analysis of genome-wide experiments in the context of a large high-throughput data compendium. This study proposed a way to analyze novel experimental data for an organism in the context of a large, heterogeneous available set of functional profiles, allowing incremental refinement of a set of characterized and annotated functional modules for that organism. Since human data was still scarce then, we demonstrated this idea on *Saccharomyces cerevisiae*, using a compendium of some 2000 experiments from 60 different publications, including gene expression profiles, transcription factor location, synthetic lethality, and protein interactions. Functional modules were found by using our SAMBA algorithm, which identifies biclusters in the compounded data.

Over the past 20 years, novel experimental and computational techniques have led to an explosion of studies on functional modules and networks in biology: A simple PubMed search reports some 7000 studies in 2005, and 51,000 in 2024. These include mostly biological findings but also many new analysis methods. Novel omics assays and more recently single-cell assays enable analyses at a highly detailed level, and multi-omic integration of such data is still a major challenge. In parallel, extensive repositories of experimental profiles are maintained by the community and enable deep analysis. While our 2005 vision of a continuously updated repository of functional modules (biclusters) has not materialized on a large scale, excellent extensive databases on pathways, networks, protein complexes are maintained, and integrative tools for their analysis continue to be developed and updated. Still, effective analysis of multiple data modalities beyond 2-5 types of data remains a challenge.

Functional modules are a powerful paradigm for organizing and interpreting biological mechanisms and dynamics. However, a major challenge in realizing their potential to advance disease understanding and improve healthcare and societal well-being lies in the limited access to large-scale clinical data. Unlike the relative openness of omics, pathway, and cellular data, patients’ electronic medical records remain siloed across the globe. The field of systems biology can play a pivotal role in bridging functional and clinical analysis by advocating for and insisting on the open sharing of anonymized medical records. Such efforts would not only enhance translational research but also deepen our understanding of the fundamental principles of biology.

## Dennis Vitkup


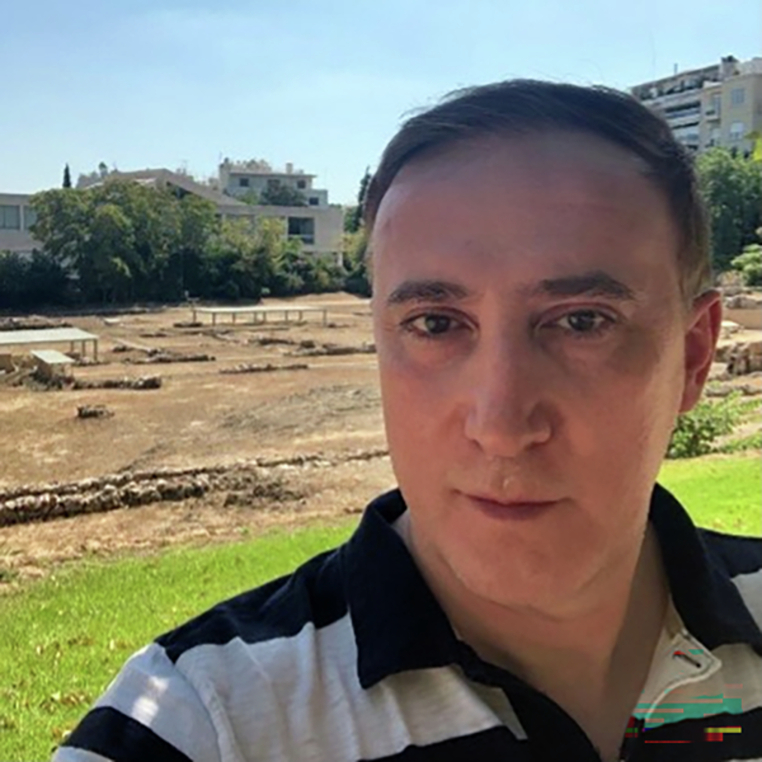


Our publication in the inaugural *MSB* issue provided one of the first in-depth analyses of the local and global regulation of cellular metabolic networks. Using the yeast whole-genome network as an example, we demonstrated multiple patterns of regulation at the levels of single proteins, structural network motifs, distinct biochemical pathways, and the global metabolic network. Similar regulatory patterns have since been discovered in the metabolic networks of many other organisms.

### History of systems biology: the past 20 years

In my opinion, systems biology as a field has made remarkable progress over the past 20 years. For example, among the major computational advances are the development of multiple constraint-based methodologies for modeling the metabolism of thousands of species, as well as methods for the reconstruction and analysis of whole-cell regulatory, interaction, and signaling networks. Researchers have also identified networks and cell types primarily affected in cancer, metabolic, immune, psychiatric, and neurodegenerative disorders. On the experimental side, amazing progress has been achieved in single-cell and high-resolution spatial sequencing technologies, which have revealed comprehensive cell-type atlases for humans and other organisms.

### What is systems biology?

In my view, the central question of systems biology is how biological functions and organismal traits emerge from interactions within complex biological systems. Similar to other fields, such as biochemistry and molecular biology, the approaches and conceptual frameworks of systems biology have been adopted by many other disciplines, including immunology, neuroscience, microbiology, and evolutionary biology. This does not prevent systems biology from being a stand-alone field; rather, it is a testament to the importance of systems biology thinking in addressing fundamental biological questions in multiple areas.

### Current frontiers of systems biology

Looking ahead, I believe the future of systems biology lies, first, in unifying knowledge-based (AI) and mechanistic-based approaches to gain mechanistic understanding and to improve predictive abilities of various biomedical applications. Second, it lies in the multiscale integration of methods across molecular, network, cellular, organismal, and population levels of biological organization.

